# Copper-Induced In Vivo Gene Amplification in Budding Yeast

**DOI:** 10.34133/bdr.0030

**Published:** 2024-03-28

**Authors:** Junyi Wang, Jingya Song, Cong Fan, Jiahao Duan, Kaiyuan He, Jifeng Yuan

**Affiliations:** ^1^ State Key Laboratory of Cellular Stress Biology, School of Life Sciences, Faculty of Medicine and Life Sciences, Xiamen University, Fujian 361102, China.; ^2^ Key Laboratory for Synthetic Biotechnology of Xiamen City, Xiamen University, Fujian 361005, China.; ^3^ Shenzhen Research Institute of Xiamen University, Shenzhen 518057, China.

## Abstract

In the biotechnological industry, multicopy gene integration represents an effective strategy to maintain a high-level production of recombinant proteins and to assemble multigene biochemical pathways. In this study, we developed copper-induced in vivo gene amplification in budding yeast for multicopy gene expressions. To make copper as an effective selection pressure, we first constructed a copper-sensitive yeast strain by deleting the *CUP1* gene encoding a small metallothionein-like protein for copper resistance. Subsequently, the reporter gene fused with a proline–glutamate–serine–threonine-destabilized *CUP1* was integrated at the δ sites of retrotransposon (Ty) elements to counter the copper toxicity at 100 μM Cu^2+^. We further demonstrated the feasibility of modulating chromosomal rearrangements for increased protein expression under higher copper concentrations. In addition, we also demonstrated a simplified design of integrating the expression cassette at the *CUP1* locus to achieve tandem duplication under high concentrations of copper. Taken together, we envision that this method of copper-induced in vivo gene amplification would serve as a robust and useful method for protein overproduction and metabolic engineering applications in budding yeast.

## Introduction

The field of synthetic biology aims to engineer biological systems for a wide range of applications, including the production of recombinant proteins [1,2] and biochemicals [3–7]. Designing bioprocesses in microbial cell factories is widely recognized as an effective means to achieve economically viable levels of high-value products [8–12]. *Saccharomyces cerevisiae*, a generally recognized as safe microorganism, is considered as an industrial workhorse for chemical and recombinant protein synthesis [13–19], owing to its biosafety status, robust growth properties, and the availability of extensive genetic engineering tools. The efficient utilization of yeast cell factories often necessitates metabolic engineering adjustments in various biological fractions [20–22]. To decouple the growth and production phase, a variety of controllable expression systems have been established in budding yeast. For instance, Zhou et al. [23] reported the temperature-sensitive Gal4 mutant Gal4M9 for constructing a temperature-dependent regulatory system. By refactoring the galactose-regulated (GAL) system, it can respond to copper ions [24,25], cyanamide [26] and cell population [27].

A number of studies revealed that marked impacts on product synthesis by the manipulation of protein expression levels at the gene copies [28–30]. In budding yeast, researchers commonly use the low-copy centromeric plasmids and high-copy 2μ plasmids for proof-of-concept studies. To maintain these plasmids in cells, it necessitates the application of auxotrophic or antibiotic selection markers [31]. However, the plasmid loss over a prolonged fermentation period causes the copy number instability [32], leading to heightened fermentation costs and unstable protein expression levels [33]. To improve the genetic stability of plasmid-based expression systems, the glycolytic related genes such as *TPI1* gene was used as a selection marker to complement the ∆*tpi1* yeast strain [34]. When compared to the plasmid-based system, chromosome-based gene expressions are more favorable alternatives for industrial applications because of the enhanced genetic stability. To rapidly assemble complex biochemical pathways and to achieve an efficient expression of metabolic modules in yeast, chromosomal gene integration with multicopies is much preferred [35–37]. In budding yeast, 2 repetitive sequences, namely, the δ sites of retrotransposon (Ty) elements and the ribosomal DNA (rDNA) region, are commonly used as integration sites for protein overexpression and metabolic engineering applications [29,38–41]. These repetitive sites allow for gene dosage augmentation or the creation of engineered strains harboring multiple metabolic modules.

**Fig. 1.**
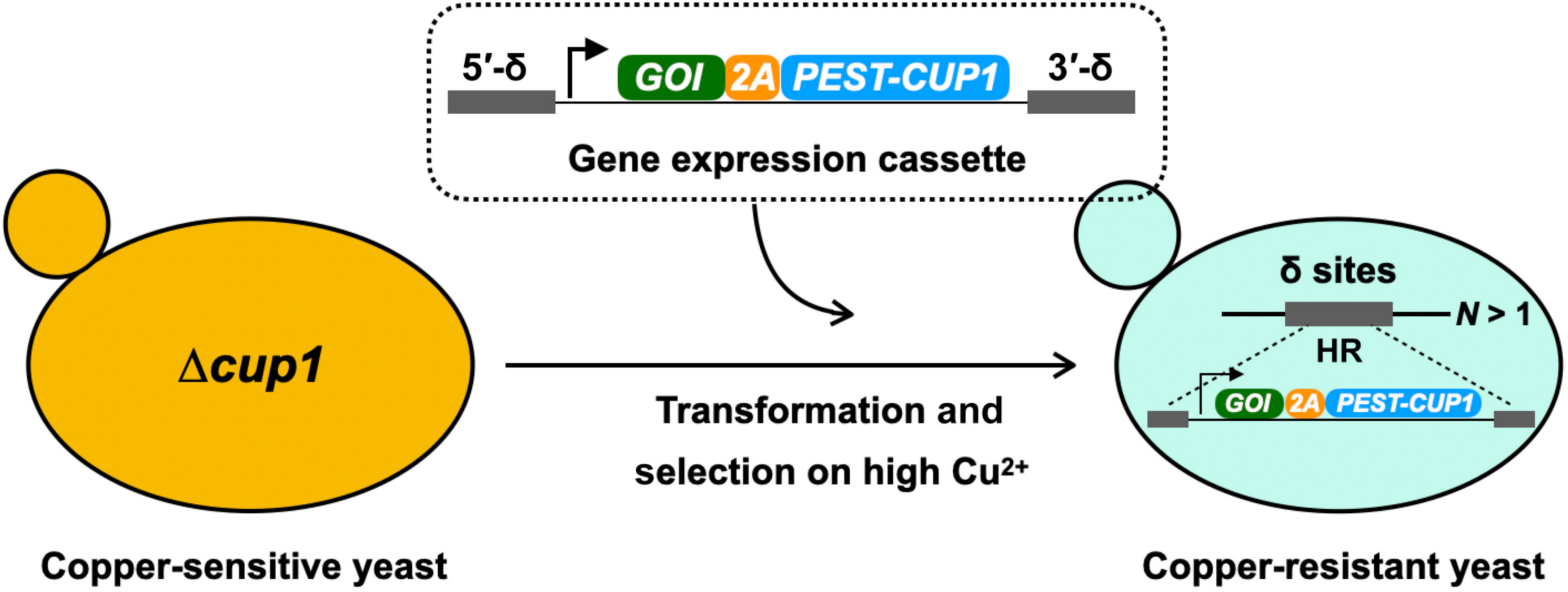
Schematic diagram of CUP1-mediated δ integration for in vivo gene amplification in budding yeast. The GOI expression cassette is generated by tandemly linking the POI to a self-cleavable 2A peptide (GSGATNFSLLKQAGDVEENPGP), a PEST degron, and the *CUP1* gene (encoding a relatively small metallothionein-like protein with 61 amino acids). To enable multicopy gene integration at the δ sites of Ty elements, high concentrations of copper ions are applied to evolve the yeast genome so that *N* for individual cassette is >1. HR, homologous recombination.

The trace element of copper plays crucial roles in microorganisms, and its homeostasis in *S. cerevisiae* has been well documented [42–44]. In this study, we report that the endogenous *CUP1* gene encoding a small metallothionein-like protein responsible for copper resistance [45] could serve as a useful selection marker for in vivo gene amplification in yeast. As shown in Fig. 1, the protein of interest (POI) was fused with a self-cleavable 2A peptide [46,47] and a proline–glutamate–serine–threonine (PEST) [48] destabilized CUP1 protein. We demonstrated that the acquisition of varying copy numbers of the expression cassette at the repetitive δ sites could be achieved in the yeast that was engineered to make it susceptible to copper. In addition, we also examined the genetic stability of the engineered strains with multicopy integration remained very stable after successive rounds of passaging. Furthermore, a simplified design of tandem duplication at the *CUP1* locus could be also achieved by applying high concentration of Cu^2+^ selection. Taken together, we established an alternative and efficient approach using copper-induced in vivo chromosomal evolution for multicopy integration in budding yeast, which is expected to accelerate the production of recombinant proteins and value-added compounds with enhanced efficiency and scalability.

## Materials and Methods

### Strains and reagents

*Escherichia coli* strain TOP10 was used for general plasmid constructions, and the strain was maintained at 37 °C in Luria–Bertani medium [tryptone (10 g/l), yeast extract (5 g/l), and NaCl (10 g/l)]. Strain JY-Cyan with a cyanamide-inducible GAL system [26] was used as the starting yeast strain for further genetic modifications. The engineered yeast strains were cultured at 30 °C in the yeast–peptone–dextrose (YPD) medium [tryptone (20 g/l), yeast extract (10 g/l), and glucose (20 g/l)] or in the yeast–nitrogen–base–glucose (YNBD) medium with appropriate dropouts. Enzymes (high-fidelity Phusion polymerase, *Sac*I-HF, *Bam*HI-HF, *Xho*I-HF, *Bsa*I-HF, and T4 DNA ligase) were purchased from New England Biolabs (Ipswich, MA, USA). Polymerase chain reaction (PCR) purification kit, gel extraction kit, and plasmid DNA extraction kit were all purchased from BioFlux (Shanghai, China). The chemicals used in this study were obtained from Sigma-Aldrich or otherwise stated.

### Construction of plasmids and strains

The oligonucleotides used in this study are listed in Table S1. All the DNA fragments were PCR-amplified using high-fidelity Phusion DNA polymerase. The in-house designed plasmid pRS425-GGA [49] was used as the backbone for construction of the plasmid pRS425-eGFP-2A-PEST-CUP1. First, the 2A-PEST sequence was amplified from *StyA-2A-StyB* gene [50], and the promoter sequence of P_GAL1_ and the *CUP1* gene were amplified from the genomic DNA of CEN.PK2-1C using the primer pairs listed in Table S1. Subsequently, these DNA fragments were assembled into pRS425-GGA using the *Bsa*I-mediated Golden-gate assembly method to yield pRS425Gal1-2A-PEST-CUP1. Furthermore, the enhanced green fluorescent protein (eGFP) sequence was amplified from pKT127 [51] using the primer pair of GFP_BamHI_fwd and GFP_XhoI_rev, digested with *Bam*HI/*Xho*I and inserted into pRS425Gal1-2A-PEST-CUP1 cut with the same enzymes, to obtain pRS425-eGFP-2A-PEST-CUP1. For the construction of pRS425-HR-P_GAL1_, the ~500-base-pair (bp) homologous fragment from the upstream of *CUP1* gene together with P_GAL1_ promoter was obtained by overlapping PCR, digested with *Sac*I/*Bam*HI, and inserted into the yeast expression vector pRS425Gal1. Subsequently, the *Bam*HI/*Xho*I digested eGFP fragment was inserted into pRS425-HR-P_GAL1_ to yield pRS425-HR-P_GAL1_-eGFP. All the details of plasmids are provided in Table S2.

Genome editing mediated by CRISPR-Cas9 was performed using electroporation as previously reported [38]. Briefly, 50 μl of yeast cells together with approximately 2-μg mixture of genome editing cassette was electroporated in a 0.2-cm cuvette at 1.6 kV. After electroporation, cells were immediately mixed with 5 ml of 1:1 mix of 1 M sorbitol:YPD medium and recovered in a rotary shaker for 1 h. Following that, cells were collected by centrifugation at 3,000 rpm for 5 min, washed, and resuspended in 50 μl of double-distilled H_2_O. Next, 50 μl of cells were plated on YNBD-URA-TRP plates. Randomly picked colonies were subjected to diagnostic PCR verification of genome editing events. All genetic modifications of ∆cup1, ∆gal7-10-1, ∆gal7-10-1:P_GAL1_-eGFP, P_CUP1_:CTR1, and ∆CUP1T: HR-P_GAL1_-eGFP were obtained by CRISPR-Cas9 method. To achieve multicopy integration of the expression cassette, the mutant library was typically obtained at the selection pressure of 0.1 mM Cu^2+^ and 0.1 mM cyanamide. All the details of strains are provided in Table S3.

### Measurement of green fluorescence intensity

The yeast culture was cultivated in a rotary shaking incubator at 30 °C and 250 rpm. The cell cultures were treated with 1 mM cyanamide to induce the eGFP expression under the control of GAL promoter. After 24 h of cultivation, samples were collected for measuring optical density at 600 nm (OD_600_) and eGFP intensity by the microplate reader (BioTek, Synergy H1). The eGFP intensities were monitored by setting 480 nm as the excitation wavelength and 520 nm as the emission wavelength. To exclude the interference from complex components in the culture medium, all samples were centrifugated and resuspended in double-distilled H_2_O before the measurement of eGFP values.

### Genetic stability analysis

The engineered yeast strains with multicopy integration of expression cassettes were passaged in the YPD medium to confirm the genetic stability of integrated cassettes by monitoring the expression levels of eGFP. Yeast cells were first cultured in 2 ml of YPD medium for 24 h until the cell growth reached the stationary phase. Next day, 1% of the culture was transferred to a fresh 2 ml of YPD medium. The series passaging was repeated for 6 rounds. During each round of passaging, the yeast cells were also cultivated in the YNBD medium supplemented with 1 mM cyanamide for the induction of eGFP expression. The OD_600_ and eGFP values were measured after 24 h of cultivation using the abovementioned approach.

## Results and Discussion

### Disrupting the copper hemostasis by CUP1 knockout

The increasing demand for recombinant proteins with industrial and pharmaceutical values requires multicopy expression systems for improved protein yield and productivity. The heterologous gene expressions in *S. cerevisiae* typically involve either plasmid-based systems or chromosomal integrations. In the former approach, the variation of plasmid copy numbers prevents its use for industrial applications, not to mention that plasmids may be gradually lost during fermentation, even in a selective medium [52,53]. In general, chromosomal integration of repetitive sequence loci is assessed for multicopy integration under high antibiotic selection pressure. To stabilize the inheritance of metabolic modules in industrial applications without resorting to excessive antibiotic usage, auxotrophic selection markers such as glycolytic genes can also be used [54,55]. The *CUP1* gene (encoding a metallothionein protein consisting of 61 amino acids) confers copper resistance in yeast, and it is a relatively small marker when compared to the commonly used auxotrophic or antibiotic selection markers.

**Fig. 2.**
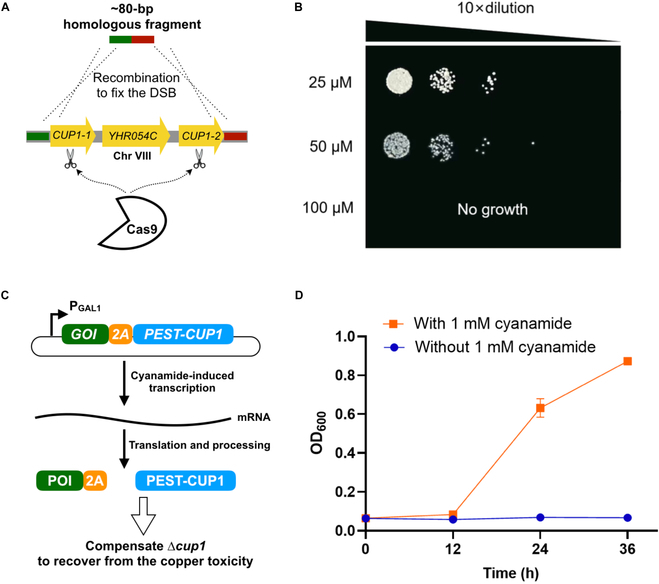
Engineering *S. cerevisiae* to make it susceptible to copper ions. (A) Schematic diagram of Cas9-mediated genome editing to knockout **CUP*1* gene. (B) The tolerance of strain JY-Cyan-∆cup1 to different concentrations of copper ions. The engineered strain JY-Cyan-∆cup1 was spotted on YNBD agar plates supplemented with 25, 50, or 100 μM Cu^2+^. Images were taken after 3 d. (C) Schematic diagram of plasmid-based expression of the *eGFP-2A-PEST-CUP1* cassette to compensate ∆*cup1*. (D) Rescuing the Cu^2+^ toxicity by the complementation of plasmid-based *CUP1* expression. Strain JY-Cyan-∆cup1 carrying a 2μ plasmid containing the expression module of eGFP-2A-PEST-CUP1 under the GAL1 promoter was cultivated in YNBD medium + 100 μM Cu^2+^. Cyanamide (1 mM) was added to trigger the expression of eGFP-2A-PEST-CUP1. Data represent the mean values with SDs from triplicate biological experiments.

*S. cerevisiae* has naturally evolved multiple copies of *CUP1* in tandem to confer the resistance against copper toxicity [56]. To enable *S. cerevisiae* susceptible to copper ions, it is necessary to abolish the native *CUP1* to prevent gene duplication occurring at the *CUP1* locus. We therefore knocked out the *CUP1* gene of JY-Cyan to obtain strain JY-Cyan-∆cup1 using the CRISPR-Cas9-mediated approach (Fig. 2A). Notably, we found that the deletion of CUP1 together with YHR054C did not give a noticeable change of yeast fitness under normal cultivations. To assess the impact of the Δcup1 on copper resistance, we conducted the growth experiments with strain JY-Cyan-∆cup1 on YNBD agar plates containing varying concentrations of copper ions (25, 50, and 100 μM Cu^2+^). As can be seen in Fig. 2B, strain JY-Cyan-∆cup1 was unable to grow on the YNBD agar plate supplemented with 100 μM Cu^2+^, indicating that the copper ion concentrations exceeding 100 μM can be used as an effective selection pressure.

### Characterization of the functionality of 2A-PEST-CUP1 tag

We next sought to harness copper ion as a cost-effective selection pressure for multigene integration at the repetitive δ sites in yeast. Specifically, the gene-of-interest (GOI) expression cassette was generated by tandemly linking the POI to a self-cleavable 2A peptide and a destabilized CUP1. It was reported that the PEST sequence is considered to be flexible, unstructured, and one of the most common motifs for protein degradation through the 26*S* proteasome pathway in mammalian cells, plants, and yeast [57]. To reduce the half-life of CUP1 and increase the selection pressure from copper, we designed a self-cleavable 2A-PEST-CUP1 and in-frame fused it to the C terminus of POI, so that the reduced half-life of CUP1 ensures multicopy integration of the expression cassette to counter the copper toxicity.

To validate that the susceptibility of engineered *S. cerevisiae* to the copper ions can be rescued by reintroducing *CUP1* expression (Fig. 2C), we constructed a 2μ plasmid containing the gene expression cassette of eGFP-2A-PEST-CUP1 and transformed it into JY-Cyan-∆cup1. Since the cyanamide-inducible GAL system is tightly regulated by cyanamide [26], we observed that the engineered strain exhibited viability only when the cyanamide inducer was added to trigger the expression of eGFP-2A-PEST-CUP1 (Fig. 2D). These findings also suggested that the design of a self-cleavable 2A-PEST-CUP1 did not prohibit the functional expression of *CUP1*, thereby conferring the resistance to copper ions after cyanamide induction.

### Multicopy integration at the δ sites by selection against high copper concentration

Upon the construction of copper-susceptible *S. cerevisiae* and the validation of functional expression of the 2A-PEST-CUP1 module, we subsequently aimed to investigate whether the gene expression cassette could be integrated at the δ sites by applying the copper selection [29,39]. Since the ability of yeast strains to resist Cu^2+^ was affected not only by the number of integrated copies but also by the expression level of *CUP1* gene, we adopted our recently constructed cyanamide-inducible GAL system [26], so that the gene expression cassette can be tightly regulated by cyanamide concentrations. This approach ensures that even a low amount of inducer could lead to much higher selection pressure for multi-integration events.

**Fig. 3.**
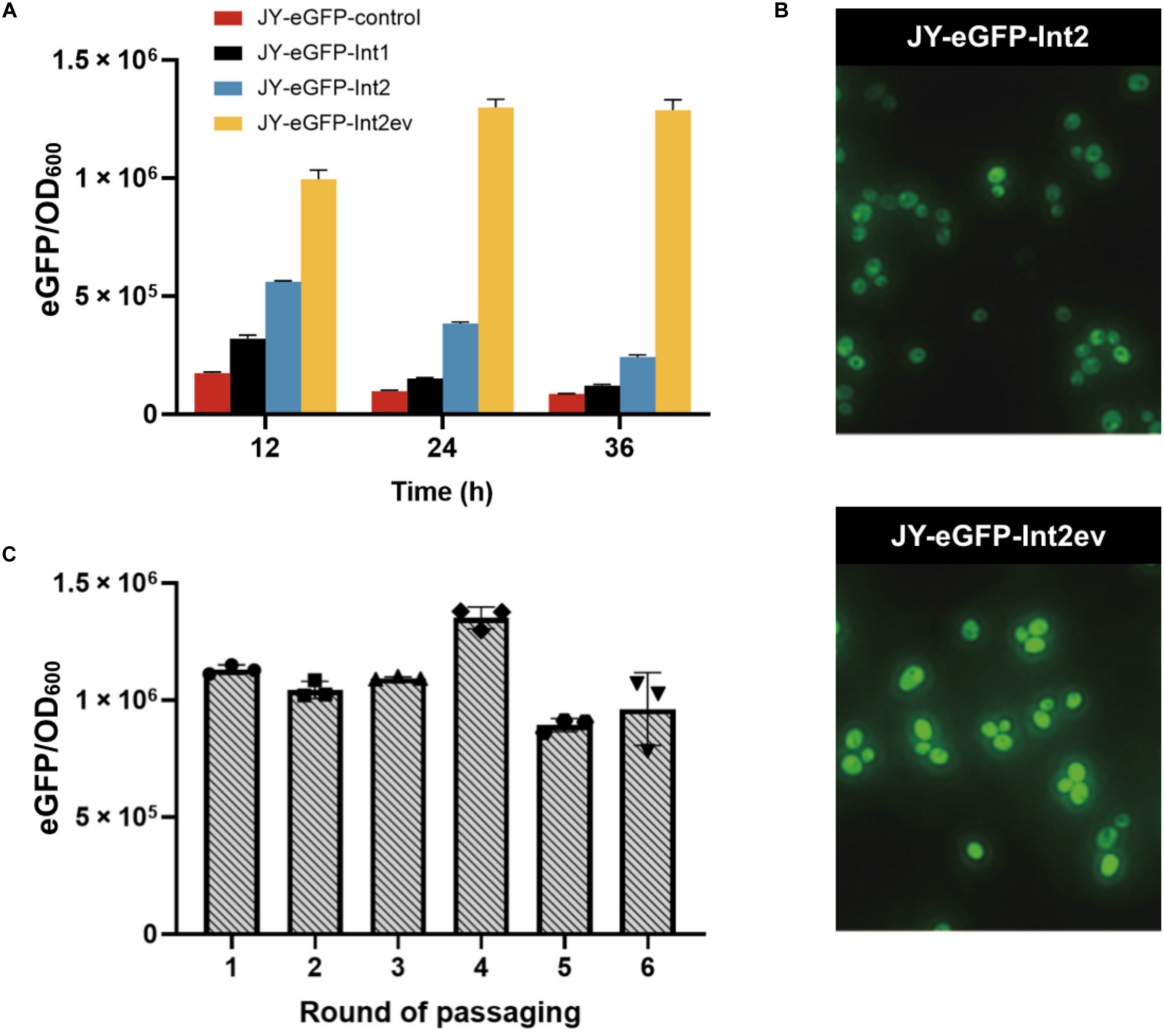
Multicopy gene integration at the repetitive δ sites via selection against copper. (A) The eGFP levels of engineered strains obtained from the library. The expression cassette of eGFP-2A-PEST-CUP1 was transformed into JY-Cyan-∆cup1*. Strain JY-eGFP-Int1 and JY-eGFP-Int2 were obtained from the library treated with 100 μM Cu^2+^ and 0.1 mM cyanamide. Strain JY-eGFP-Int2ev was obtained by evolving strain JY-eGFP-Int2 under the selection pressure of 1 mM Cu^2+^ and 0.1 mM cyanamide. (B) Fluorescence microscopy analysis of eGFP distribution profile of engineered yeast strains. Strain JY-eGFP-Int2 and JY-eGFP-Int2ev were compared under the same condition. Images were captured after 24 h of cultivation. (C) The genetic stability of engineered strain JY-eGFP-Int2ev after repeated passaging. The yeast cells were subcultured in the YPD medium every 24 h for 6 rounds of passaging. After each round of passaging, the yeast cells were induced by 1 mM cyanamide for the expression of eGFP. Both OD_600_ and eGFP values were measured after 24 h of incubation. Data represent the mean values with SDs from triplicate biological experiments.

Next, we proceeded to integrate the expression cassette of eGFP-2A-PEST-CUP1 at the δ sites in strain JY-Cyan-∆cup1* (* indicates ∆*gal7-10-1* to prevent the unwanted cyanamide-induced expression of *GAL7-10-1*) by selecting on YNBD agar plates containing 100 μM Cu^2+^ and 0.1 mM cyanamide. To compare the gene dosage effect of δ integration, we also constructed a single copy of eGFP-2A-PEST-CUP1 and integrated it into the yeast chromosome at the **GAL*7-10-1* site, and the resulting strain was designated as JY-eGFP-control. As shown in Fig. 3A, 2 randomly isolated transformants exhibited much higher eGFP/OD_600_ values compared to the reference strain JY-eGFP-control. These findings suggested that both strain JY-eGFP-Int1 and JY-eGFP-Int2 had multiple copies of the targeted gene cassette into the yeast chromosomes, whereas JY-eGFP-Int2 potentially had a higher number of integrated eGFP copies to give a better eGFP/OD_600_ output.

### Evolving chromosomal rearrangements by higher copper selection

Next, we sought to adjust the copper ion concentration to answer whether a higher selection pressure could be implemented to induce chromosomal rearrangements between the δ sites, thereby enabling the acquisition of evolvable strains. Such flexibility of design is expected to provide researchers with the capacity to optimize the production of targeted proteins or metabolites to fulfill specific applications. In particular, strain JY-eGFP-Int2 was cultivated on YNBD plates containing 0.1 mM cyanamide and 1 mM Cu^2+^ at 30 °C for 3 d. As shown in Fig. 3A, we managed to isolate a mutant strain of JY-eGFP-Int2ev that exhibited a substantial increase in eGFP/OD_600_ output compared to the parental strain JY-eGFP-Int2, and the eGFP/OD_600_ value of JY-eGFP-Int2ev was about 3.5 times over that of strain JY-eGFP-Int2. Further fluorescence microscopy analysis of engineered yeast strains revealed that the cell morphology of strain JY-eGFP-Int2ev did not change much when compared to that of JY-eGFP-Int2, whereas strain JY-eGFP-Int2ev gave uniformly higher eGFP signals over its parental strain of JY-eGFP-Int2 (Fig. 3B). These findings confirmed the feasibility of modulating chromosomal rearrangements to further increase the protein expression by subsequent strain evolution under higher concentrations of Cu^2+^. In the future, further modulating the abundance of CUP1 might be explored to reduce the required concentration of copper for selection.

The genetic stability of the engineered strains is an important parameter for the industrial production of recombinant proteins and biochemicals. Considering that the δ sites of Ty elements are highly repeated sequences, there remains in doubt whether the genetic stability of integrated cassettes could be maintained under the nonselective condition of physiological copper concentrations. To address this, we repeatedly passaged strain JY-eGFP-Int2ev in the nonselective YPD culture medium for 6 rounds (approximately 10 generations of propagation per passaging). As shown in Fig. 3C, the genetic stability of JY-eGFP-Int2ev was well maintained after 6 rounds of passaging, suggesting that the widely dispersed δ sites are relatively resilient to pop out the integrated cassettes under nonselective conditions. On the basis of the success of copper-induced chromosomal evolution, similar design principles might be explored to construct more compact expression cassettes. For instance, a weak intergenic sequence (IGG) from the filamentous fungus *Glarea lozoyensis* [58,59] could be implemented to construct a polycistronic system of GOI-IGG-CUP1, and a relatively low-level translation from IGG is expected to secure multicopy integration to counter the copper toxicity.

### A simplified design of tandem gene duplication system

**Fig. 4.**
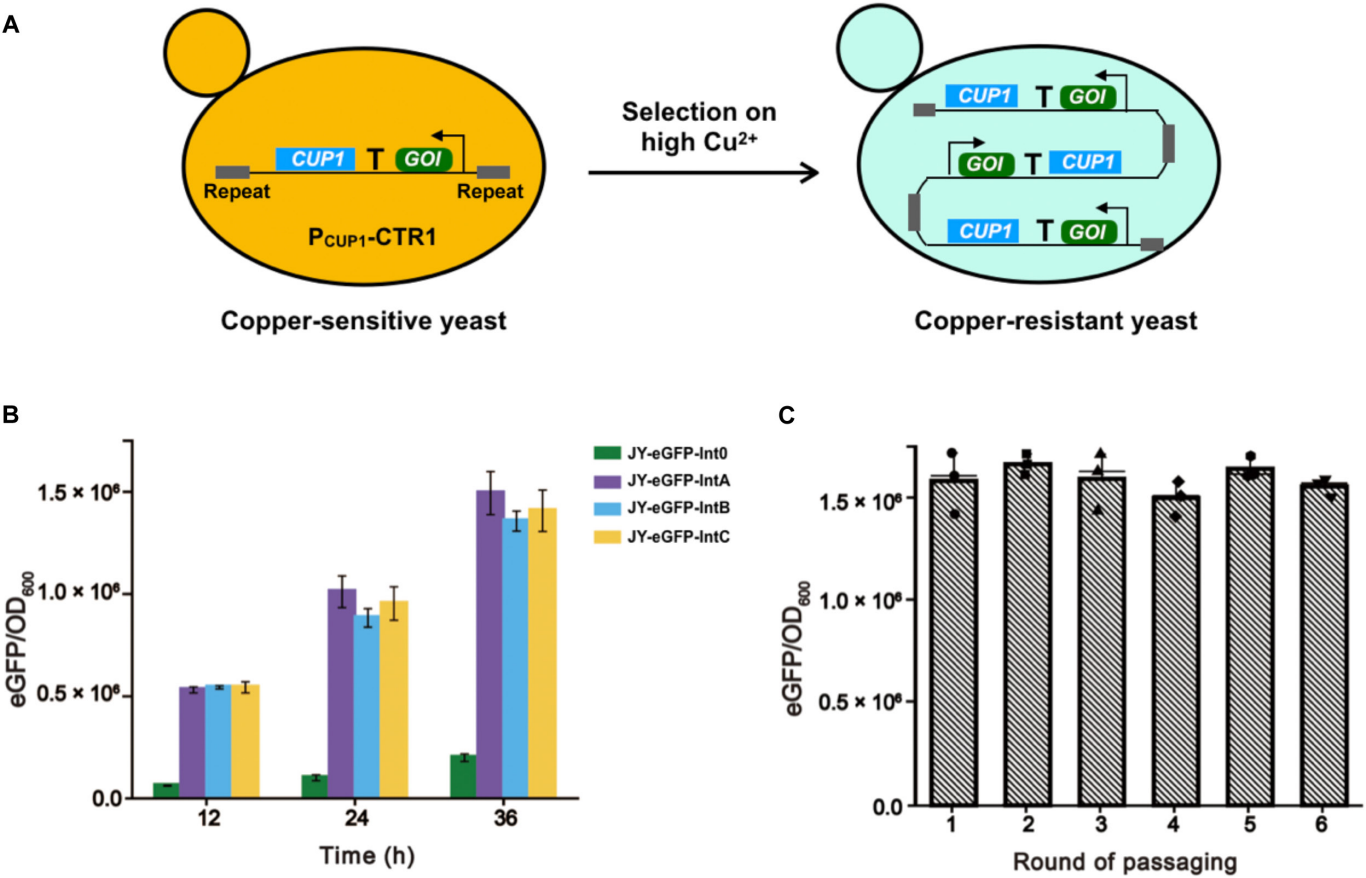
A simplified design of tandem gene duplication at the *CUP1* locus. (A) Schematic illustration of the mode of action for tandem gene duplication. P_CUP1_-CTR1 is used to avoid the reduced Cu^2+^ import. Next, the expression cassette is integrated at the *CUP1* locus with homologous repeats. Upon selection on high concentration of Cu^2+^, the GOI-CUP1 cassette is tandemly duplicated to counter the copper toxicity. (B) The eGFP levels of engineered strains obtained from the library. The mutant library was obtained by evolving strain JY-eGFP-Int0 under the selection pressure of 1 mM Cu^2+^ and 0.1 mM cyanamide. (C) The genetic stability of engineered strain JY-eGFP-IntA after repeated passaging. Experiments were carried out in a similar way as Fig. 3B. Data represent the mean values with SDs from triplicate biological experiments.

Although the feasibility of 2A-PEST-CUP1 tagging system for multicopy integration has been successfully established in budding yeast, it is not desirable for some proteins with the residue 2A peptide that might affect the protein activity. In addition, the synthesis of high-level 2A-PEST-CUP1 and the degradation of CUP1 by the 26*S* proteasome pathway are energy-consuming steps. Moreover, it remains in doubt whether the 2A-PEST-CUP1 system will be implementable for secreted proteins, as the trafficking of CUP1 during the secretion process might affect its function. We next sought to develop a tandem gene duplication system to improve the flexibility of design (Fig. 4A). As the copper transporter such as CTR1 and CTR3 are subjected to copper repression at the transcription level, we first engineered P_CUP1_-CTR1 to increase the selection pressure from Cu^2+^. Subsequently, we integrated the expression cassette at the terminator region of the *CUP1* locus with an additional upstream homologous repeat. Upon selection on high concentration of Cu^2+^, the GOI-CUP1 cassette is expected to be tandemly duplicated to increase the *CUP1* copies and to counter the copper toxicity at elevated concentrations. Notably, unlike the GOI-2A-PEST-CUP1 design, the tandem gene duplication at the *CUP1* locus does not affect the secretion process of targeted proteins, which is more desirable for future secreted protein overproduction.

As shown in Fig. 4B, several randomly picked strains from the mutant library exhibited substantially higher levels of eGFP/OD_600_ than that of JY-eGFP-Int0, indicating that tandem gene duplication is a viable approach for multicopy gene integration in yeast. We also repeatedly passaged strain JY-eGFP-IntA in the nonselective YPD medium for 6 rounds. As illustrated in Fig. 4C, the genetic stability of JY-eGFP-IntA was also maintained after 6 rounds of passaging. As the selection marker of CUP1 is independently expressed under its native promoter that is not active under nonselective conditions, it is less energy-consuming during the POI production when compared to that of the GOI-2A-PEST-CUP1 design. In comparison to the conventional antibiotic or auxotrophic selection methods, the conditional expression of *CUP1* under its native copper-inducible promoter will not deplete the cellular resources for the synthesis of selection marker, which would in theory allow more efficient production of targeted proteins.

## Conclusion

In summary, we have explored the *CUP1* gene as an effective selection marker for multicopy gene integration in budding yeast. The varying copy numbers could be adjusted by modulating the selection pressure through different concentrations of Cu^2+^. We first successfully achieved multicopy integration by GOI-2A-PEST-CUP1. Unlike the traditional δ and recombinant DNA integration that rely an additional cassette for expressing selection markers [29,38–41], our design of GOI-2A-PEST-CUP1 only requires a single pair of promoter and terminator to drive the expression of all components. In particular, further improving the protein expression was obtained by evolving chromosomal rearrangements under higher copper ion selection pressure. Moreover, we also demonstrated that the tandem duplication at the *CUP1* locus represents a simplified and universal design for the effective recombinant protein overexpression. Both methods resulted in engineered strains with relatively stable genetic constructs even in the nonselective medium, making them readily applicable for industrial applications. Taken together, copper-induced in vivo chromosomal evolution for multicopy integration in budding yeast would serve as a powerful tool for enhancing recombinant protein overproduction and constructing extensive biochemical pathways in the realms of synthetic biology and metabolic engineering researches.

## Data Availability

The data involved in the research are included in the manuscript and the Supplementary Materials. All relevant data are available upon reasonable request from the corresponding author.
