# Supplementary material for: Copper-Induced In Vivo Gene Amplification in Budding Yeast
Source: Biodes Res. 2024 Mar 28;6:0030. doi: 10.34133/bdr.0030 (PMC10976586; doi:10.34133/bdr.0030)
Supplement: Supplementary 1 — Tables S1 to S3 [file bdr.0030.f1.docx]

**Supplementary Materials**

**Copper-Induced *In Vivo* Gene Amplification in Budding Yeast**

Junyi Wang^1,#^, Jingya Song^1,#^, Cong Fan^1,#^, Jiahao Duan^1^, Kaiyuan He^1^, Jifeng Yuan^1,2,3^*****

^1^ State Key Laboratory of Cellular Stress Biology, School of Life Sciences, Faculty of Medicine and Life Sciences, Xiamen University, Fujian 361102, China

^2^ Key Laboratory for Synthetic Biotechnology of Xiamen City, Xiamen University, Fujian 361005, China

^3^ Shenzhen Research Institute of Xiamen University, Shenzhen 518057, China

^#^ These authors contributed equally to the experimental work

* Corresponding author Email: [jfyuan@xmu.edu.cn](mailto:jfyuan@xmu.edu.cn)

| **Table S1 List of oligonucleotides used in this study** | |
| --- | --- |
| Name of primers | Sequence |
| gCUP1_F | TTGGTCTCAGATGTATCAGCCTGAAATAAAGGGGTTTTAGAGCTAGAAATAG |
| gGAL1CDS_F | TTGGTCTCAGATGAGCAACGGCACAAATGAATGGTTTTAGAGCTAGAAATAG |
| gCUP2T_F | TTGGTCTCAGATGATACGTTAATAATTAAGAAAGTTTTAGAGCTAGAAATAG |
| SUP4t_R | TTGGTCTCAAAAGAGACATAAAAAACAAAAAAAG |
| CUP1_Del_F | ATCCCATTACCGACATTTGGGCGCTATACGTGCATATGTAACGAATAGTC |
| CUP1_Del_R | CAGCAAATAGTTAGATGAATATATTAAAGACTATTCGTTACATATGCACG |
| CUP1_up | CGATCCCATTACCGACATTTG |
| CUP1_down | GTTAGGCAAACTAGAATTTGG |
| Gal7-10-1_Del_F | TTCTTTTTACAGTCTTTGTAGATAATGAATCTGACCATCTCTAAACCAG |
| Gal7-10-1_Del_R | TTATAATTCATATAGACAGCTGCCCAATGCTGGTTTAGAGATGGTCAGA |
| Gal7-10-1_Int_F | GAGAAGTTGTTCTGAACAAAGTAAAAAAAAGAAGTATACACGGATTAGAAGCCGCCGAG |
| Gal7-10-1_Int_R | TAGAAAAAATATGATATGAATGAATATTCCACTTTCTTTCTTCGAGCGTCCCAAAACC |
| Gal1_R100 | GTAGTTGAAGCATGTATGAAC |
| pGAL1_S1_fwd | TTGGTCTCAATCTACGGATTAGAAGCCGCCGAG |
| pGAL1_S1_rev | TTGGTCTCACTCGAGCATTGTTTTGGATCCTCCTTGACGTTAAAGTATAG |
| 2APEST_S2_fwd | TTGGTCTCACGAGGGTTCTGGAGCAACCAACTTTTC |
| 2APEST_S2_rev | TTGGTCTCACCATAGTAGATTCTGGAGTAGATTCAGGTCCGGGATTCTCTTCG |
| CUP1_S3_fwd | TTGGTCTCAATGGGATTCAGCGAATTAATTAACTTC |
| CUP1_S3_rev | TTGGTCTCATCTTATTTCCCAGAGCAGCATG |
| GFP_BamHI_fwd | CGGGATCCAAAACAATGTCTAAAGGTGAAGAATTATTC |
| GFP_XhoI_rev | ACGCCGCTCGAGTTTGTACAATTCATCCATACC |
| Delta_Int_fwd | TGTTGGAATAGAAATCAACTATCATCTACTAACTAGTATACGGATTAGAAGCCGCCGAG |
| Delta_Int_rev | GAGAAATGGGTGAATGTTGAGATAATTGTTGGGATTCCATCTTCGAGCGTCCCAAAACC |
| HRegion_SacI_fwd | AGAGAGAGCTCGCATCATAGAAATCGTTGAAG |
| HRegion_OE_rev | AAAGCTAGTAGCACATTTTTTACTCAATAAC |
| Pgal1_OE_fwd | GTTATTGAGTACGGATTAGAAGCCGCCGAG |
| Pgal1_BamHI_rev | CGGGATCCGGGGTTTTTTCTCCTTGACG |
| CUP1T_Int_fwd | TTTTATTCGAAATCTGGGGATTCTATACAGAGTTGTAAGCATCATAGAAATCGTTGAAG |
| CUP1T_Int_rev | CGAATAGTCTTTAATATATTCATCTAACTATTTGCTGTTCTTCGAGCGTCCCAAAACC |

| **Table S2 List of plasmids used in this study** | | |
| --- | --- | --- |
| Name | Description | Reference |
| p414-TEF1p-Cas9 | Plasmid harboring the Cas9 expression cassette | (DiCarlo et al., 2013) |
| pRS426SNR52 | pESC-URA derivative with the P_SNR52_-T_SUP4_ cassette | Lab stock |
| pgCUP1p | pRS426SNR52 derivative with gRNA targeting at the promoter region of *CUP1* | This study |
| pgGAL1CDS | pRS426SNR52 derivative with gRNA targeting at the open reading frame of *GAL1* | This study |
| pRS425GGA | Plasmid for the golden-assembly of targeted genes under the control of P_GAL1/10_ | (Yuan, Mo, & Fan, 2021) |
| pRS425Gal1-2A-PEST-CUP1 | pRS425GGA derivative with an insertion of P_GAL1_-2A-PEST-CUP1 | This study |
| pRS425-eGFP-2A-PEST-CUP1 | pRS425Gal1-2A-PEST-CUP1 derivative with eGFP-2A-PEST-CUP1 under the control of P_GAL1_ | This study |
| pRS425Gal1 | The yeast expression vector | Lab stock |
| pRS425-HR-P_GAL1_ | pRS425Gal1 derivative with HR-P_GAL1_ | This study |
| pRS425-HR-P_GAL1_-eGFP | pRS425Gal1 derivative with HR-P_GAL1_-eGFP | This study |

| **Table S3 List of strains used in this study** | | |
| --- | --- | --- |
| Name | Description | Reference |
| *E. coli* Top10 | For cloning purpose | Invitrogen |
| CEN.PK2-1C | MATa; his3D1; leu2-3_112; ura3-52; trp1-289; MAL2-8c; SUC2 | Euroscarf |
| JY-Cyan | Strain CEN.PK2-1C derivative with Δ*gal80* and *Gal4* under the control of P_DDI2_ promoter | (Song et al., 2023) |
| JY-Cyan-∆cup1 | Strain JY-Cyan derivative with ∆*cup1* | This study |
| JY-Cyan-∆cup1-C1 | Strain JY-Cyan-∆cup1 derivative with plasmid pRS425-eGFP-2A-PEST-CUP1 | This study |
| JY-Cyan-∆cup1* | Strain JY-Cyan-∆cup1 derivative with ∆*gal7-10-1* | This study |
| JY-eGFP-control | Strain JY-Cyan-∆cup1 derivative with ∆*gal7-10-1*::P_GAL1_-eGFP-2A-PEST-CUP1 | This study |
| JY-eGFP-Int1~2 | Strain JY-Cyan-∆cup1* with random integration of P_GAL1_-eGFP-2A-PEST-CUP1 at the δ sites | This study |
| JY-eGFP-Int2ev | Strain JY-eGFP-Int2 derivative obtained under high selection pressure of 1 mM Cu^2+^ | This study |
| JY-Cyan-CTR1* | Strain JY-Cyan derivative with ∆*gal7-10-1* and P_CUP1_-CTR1 | This study |
| JY-eGFP-Int0 | Strain JY-Cyan-CTR1* derivative with integration of HR-P_GAL1_-eGFP at the *CUP1* locus | This study |
| JY-eGFP-IntA~C | Strain JY-eGFP-Int0 derivative obtained under high selection pressure of 1 mM Cu^2+^ | This study |

**References**

DiCarlo, J. E., Norville, J. E., Mali, P., Rios, X., Aach, J., & Church, G. M. (2013). Genome engineering in Saccharomyces cerevisiae using CRISPR-Cas systems. *Nucleic Acids Res, 41*(7), 4336-4343.

Song, J., Fan, J., Fan, C., He, N., Ye, X., Cao, M., & Yuan, J. (2023). A Layered Genetic Design Enables the Yeast Galactose Regulon to Respond to Cyanamide. *ACS Synth Biol, 12*(9), 2783-2788.

Yuan, J., Mo, Q., & Fan, C. (2021). New Set of Yeast Vectors for Shuttle Expression in Escherichia coli. *ACS Omega, 6*(10), 7175-7180.
